# Fatigue and resting-state functional brain networks in breast cancer patients treated with chemotherapy

**DOI:** 10.1007/s10549-021-06326-0

**Published:** 2021-07-14

**Authors:** Biniam Melese Bekele, Maryse Luijendijk, Sanne B. Schagen, Michiel de Ruiter, Linda Douw

**Affiliations:** 1 Department of Anatomy and Neurosciences, Amsterdam Neuroscience, Cancer Center Amsterdam, Amsterdam University Medical Centers, Vrije Universiteit Amsterdam, Amsterdam, The Netherlands; 2 Department of Psychosocial Research and Epidemiology, Netherlands Cancer Institute, Amsterdam, The Netherlands; 3 Brain and Cognition, Department of Psychology, University of Amsterdam, Amsterdam, The Netherlands

**Keywords:** Breast cancer, Fatigue, Network neuroscience, Functional connectivity, Graph theory

## Abstract

**Purpose:**

This longitudinal study aimed to disentangle the impact of chemotherapy on fatigue and hypothetically associated functional brain network alterations.

**Methods:**

In total, 34 breast cancer patients treated with chemotherapy (BCC +), 32 patients not treated with chemotherapy (BCC −), and 35 non-cancer controls (NC) were included. Fatigue was assessed using the EORTC QLQ-C30 fatigue subscale at two time points: baseline (T1) and six months after completion of chemotherapy or matched intervals (T2). Participants also underwent resting-state functional magnetic resonance imaging (rsfMRI). An atlas spanning 90 cortical and subcortical brain regions was used to extract time series, after which Pearson correlation coefficients were calculated to construct a brain network per participant per timepoint. Network measures of local segregation and global integration were compared between groups and timepoints and correlated with fatigue.

**Results:**

As expected, fatigue increased over time in the BCC + group (*p* = 0.025) leading to higher fatigue compared to NC at T2 (*p* = 0.023). Meanwhile, fatigue decreased from T1 to T2 in the BCC − group (*p* = 0.013). The BCC + group had significantly lower local efficiency than NC at T2 (*p* = 0.033), while a negative correlation was seen between fatigue and local efficiency across timepoints and all participants (T1 rho = − 0.274, *p* = 0.006; T2 rho = − 0.207, *p* = 0.039).

**Conclusion:**

Although greater fatigue and lower local functional network segregation co-occur in breast cancer patients after chemotherapy, the relationship between the two generalized across participant subgroups, suggesting that local efficiency is a general neural correlate of fatigue.

**Supplementary Information:**

The online version contains supplementary material available at 10.1007/s10549-021-06326-0.

## Introduction

Breast cancer is the most prevalent cancer in women and the second most commonly occurring cancer overall [1]. Treatments like chemotherapy are associated with significant side effects, ranging from acute symptoms such as vomiting and diarrhea [2] to long-term systemic complications such as cardiac toxicity, fatigue and cognitive decline [3–5]. Fatigue is a subjective feeling of exhaustion or tiredness described in terms of mental capacity, perceived energy, and psychological function [6]. Studies indicate that one in four breast cancer patients suffer from severe fatigue [7]. A major identified risk factor for severe fatigue is chemotherapy, where more than 50% of patients continue to suffer from fatigue months or even years after completion of chemotherapy [8]. However, the pathogenesis of cancer-related fatigue is not well understood, and the roles of several biochemical, physiological and psychological factors have been hypothesized. Differences in brain metabolites between fatigued and non-fatigued breast cancer survivors indicate the neurobiological changes associated with fatigue [9]. Furthermore, the close association of fatigue with symptoms such as cognitive complaints, poor sleep and depression highlights the involvement of the brain in fatigue pathogenesis in breast cancer patients [10].

Several studies have described neurotoxicity associated with chemotherapy agents. Neuroimaging studies have characterized diffuse structural changes in the brain following chemotherapy, such as significant white matter integrity changes and widespread abnormalities in gray matter volume [11–16]. Task-based functional MRI (fMRI) has demonstrated functional abnormalities in brain areas associated with cognitive function [17, 18]. Over the past decade, resting-state fMRI (rsfMRI) has been providing a reliable, non-invasive method of measuring the spontaneous or intrinsic activity of the brain by measuring the blood oxygen level-dependent (BOLD) signal. Studies have described the widespread abnormalities in resting-state networks of breast cancer survivors, highlighting chemotherapy-related functional changes in the brain [19, 20]. An association between intrinsic brain connectivity and self-reported fatigue has been reported, where enhanced connectivity between the frontal gyrus and several brain regions involved in self-referential thinking was seen [21]. Furthermore, greater left inferior parietal lobule to superior frontal gyrus connectivity was observed in the fatigued subgroup, which was associated with greater physical fatigue and poorer sleep quality.

More recently, a graph theory-based approach towards analyzing functional connectivity has gained more attention. Graph theory represents the brain as a graph consisting of nodes and edges, where nodes indicate anatomical elements and edges indicate the connectivity between them. In contrast to previously available analytic methods, it allows the visualization of connectivity patterns and quantitative characterization of global organization of the brain [22]. Among others, graph theory employs topological parameters such as functional integration and segregation to describe the properties of brain networks [23]. Segregation describes the degree to which network elements form specialized communities while integration refers to the ability to combine distributed information. Measures for integration include characteristic path length and global efficiency, while segregation can be quantified using the clustering coefficient and local efficiency [24, 25]. Their relevance in investigating how brain networks relate to normal development [26, 27], aging [28] and a wide range of symptoms associated with brain disorders [26, 29, 30] has been well-documented.

A few studies have employed rsfMRI and graph theory to characterize the topological organization of the brain in patients with breast cancer. Bruno et al. [31] showed a disruption in both regional and global network properties, signaling reduced efficiency of information transfer observed in breast cancer patients following chemotherapy [31]. The breast cancer group demonstrated significantly decreased global clustering as well as marginally decreased path length compared to healthy controls. Disrupted regional network characteristics such as nodal degree (the number of connections of a region) and hub locations were also observed in frontal, striatal and temporal areas of the brain. Xuan et al. [32] demonstrated an abnormal organization of large-scale functional brain networks in chemotherapy-treated breast cancer patients with cognitive impairment [32]. The breast cancer group had lower global and local efficiency compared with healthy controls. Local alterations such as higher nodal degree and functional connectivity were seen in several prefrontal, occipital and parietal regions. They also showed that the alterations in the network were correlated to the memory deficits observed in these group of patients. However, all of the studies so far have not been able to disentangle whether breast cancer pathogenesis or the chemotherapy was responsible for such network changes. Several of the studies were cross-sectional by design and only used healthy controls for comparison. In addition, most of them lacked a baseline characterization of the brain network prior to chemotherapy. Finally, none of these studies explored whether network alterations form a neural correlate of fatigue in these patients.

This longitudinal study investigated fatigue and brain network topology related to chemotherapy. Changes in network topology and fatigue from pre-treatment to post-treatment time points were compared between breast cancer patients who received chemotherapy, breast cancer patients who did not, and non-cancer controls. Both graph measures of integration and segregation were used to allow a comprehensive overview and enable the comparison of our results with previous studies. We hypothesized that chemotherapy-treated breast cancer patients would be more fatigued than the other groups. Moreover, we expected lower segregation and integration in those patients, and these network differences and changes to correlate with greater and increasing fatigue.

## Methods and materials

### Participants

Participants of the study were breast cancer patients who had undergone surgery under general anesthesia (BC) and age-matched controls with no history of breast cancer or chemotherapy (NC). BC patients were either scheduled to receive anthracycline-based chemotherapy with or without endocrine treatment (BCC +) or did not require chemotherapy or endocrine treatment (BCC −). Participants were eligible if the following criteria were met: female, under 70 years of age, no previous history of malignancy, a formal diagnosis of primary breast cancer without distant metastases and good command of the Dutch language. Participants scheduled to receive trastuzumab following chemotherapy were excluded from the study due to the longer duration of treatment in this patient group. Controls were recruited via participants and advertisements in participating hospitals. Only those participants who attended both time points and were scanned with the same MRI scanner at each time point were included. Following surgery, all BCC + participants received anthracycline-based chemotherapeutic agents (see Supplementary Table 1 for specific treatment regimens).

The study was approved by the Institutional Review Board of the Netherlands Cancer Institute, serving as the central ethical committee for all participating institutes. Written informed consent was obtained and the study was conducted according to the principles expressed in the Declaration of Helsinki and following institutional guidelines. The scanning was performed at the Academic Medical Center of the University of Amsterdam and the Spinoza Centre for Neuroimaging.

### Data collection

All patients with breast cancer had undergone surgery under general anesthesia before inclusion (wide local excision or mastectomy), and baseline data (T1) were collected after surgery but before the start of adjuvant treatment. Follow-up assessment (T2) was done 6 months after the last cycle of chemotherapy in the BCC + group and at matched intervals in the other two groups.

Data was collected using patient-reported outcome questionnaires (PROs), neuropsychological assessments, and an MRI protocol. Longitudinal analyses of the task-related fMRI, cognitive performance and the correlation of cognitive impairment with psychological and social factors have been reported before [33–35]. The current study focuses on the analysis of the rsfMRI data and fatigue.

Fatigue was assessed using the European Organization for Research and Treatment of Cancer (EORTC) Quality of Life Questionnaire C-30 (QLQ-C30) fatigue subscale [36]. The EORTC QLQ-C30 assesses symptoms and functioning in cancer patients. It is a self-administered questionnaire with 30 symptom-focused questions scored on a numeric scale of 4 points from 1 (“not at all”) to 4 (“very much”). Scores are computed into five functional scales and multi-item symptom scales including fatigue, nausea, vomiting and pain. Furthermore, the Hopkins Symptom Checklist-25 (HSCL-25) was used to asses anxiety and depression, while the perceived stress score (PSS) was used to assess stress. Intelligence Quotient (IQ) was estimated using the Dutch version of the National Adult Reading Test (NART) [37].

### MRI acquisition and preprocessing

MRI data were acquired using a 3 Tesla Intera full-body MRI scanner (Academic Medical Center) and a 3 Tesla Achieva full-body MRI scanner (Spinoza Centre for Neuroimaging) (both Philips Medical Systems, Best, The Netherlands). A SENSE 8-channel receiver head coil was used at both locations. Functional MRI acquisition was based on T2 weighted gradient echo-planar imaging (EPI) of 38 axial slices (voxel size 2.3 × 2.3 × 2.3 mm, interslice gap 0 mm, matrix size 96 × 96, TR = 2.1 s, TE = 25 ms). 180 volumes of rsfMRI were obtained with participants lying in the scanner relaxed with their eyes open.

The FMRIB software library (FSL v5.0.9) [38] was used for pre-processing of the structural and functional images. The structural scan was segmented into gray and white matter as well as cerebrospinal fluid using BET [39], after which the Automated Anatomical Labeling Atlas (AAL atlas) [40], which contains 78 cortical regions, was co-registered to this native space. FSL-FIRST was additionally used to acquire 12 subcortical regions (excluding the right and left nuclei accumbens), together forming the 90 nodes of the functional brain network.

Preprocessing of the rsfMRI included (a) brain tissue extraction using BET [39], (b) motion correction using McFLIRT [41] (c) spatial smoothing at 5 mm, (d) a high pass filter above 0.01 Hz, (e) alignment (registration) of participants rsfMRI data to MNI space using FLIRT and FNIRT [42], (f). extracting and regressing out of cerebrospinal fluid and white matter signals from the total rsfMRI signal. Furthermore, ICA-AROMA (Independent Component Analysis-Automatic Removal of Motion Artifacts) [43] was applied to remove residual motion artifacts. Finally, the average time series across all voxels within each of the 90 atlas regions were extracted for each participant.

### Connectivity and network analysis

Pearson correlations were performed on the time series, resulting in a 90 × 90 connectivity matrix per participant. Absolute values of correlations were used, since the differential meaning of negative correlations is unknown. Calculated graph measures were normalized by dividing them with the corresponding graph values derived from 100 random networks. Random networks with identical numbers of nodes, edges and weight distribution were generated for each participant [44]. Weighted matrices were used, in light of recent findings [45], which suggest that weighted networks have overall higher test–retest and overall reliability in regional nodal efficiency compared to binarized networks.

Four graph measures were calculated to characterize the brain network’s topological organization. The clustering coefficient reflects the prevalence of clustered connectivity around individual nodes. The average clustering coefficient (*C*_p_) generalized for weighted networks can be defined as the geometric mean of link weights associated with a node [46]. The characteristic path length (*L*_p_) is equal to the average of the weighted path lengths between nodes in a network [25]. Global efficiency (*E*_glob_) is the average inverse weighted path length. Local Efficiency (*E*_loc_) of a network is equal to average efficiency of the local subgraphs. A generalization of these measures for weighted networks is given in Rubinov and Sporns [25].

All computations of graph measures were performed using the Brain Connectivity Toolbox (http://www.brain-connectivity-toolbox.net) [25] using MATLAB (R2019a, version 9.6.0, The Mathworks Inc., Natick, Massachusetts). The BrainNet Viewer (http://www.nitrc.org/projects/bnv/) [47] was used for visualization of networks.

### Statistical analysis

Differences in demographic, clinical variables, PROs and graph measures were analyzed using SPSS 25 (IBM, Armonk, NY) by means of one-way ANOVAs, *X*^2^ tests or *t*-tests as appropriate. Changes in graph measures and fatigue scores across the two time points were analyzed by repeated measures ANOVAs. No correction was performed for testing of multiple network measures. The level of significance was set at *p* < 0.05.

Given that EORTC QLQ-C30 fatigue scores are ordinal data, associations with graph measures were computed using Spearman’s correlation coefficients. Correlations between fatigue scores and potential covariates were also calculated, since previous literature has described the overlap between the symptoms of fatigue with other psychological disorders such as stress, anxiety, depression [48] and estrogen deprivation [49] especially in postmenopausal women. Thus, HSCL scores of anxiety and depression, PSS scores, menopausal status and age at measurement were used as covariates when analyzing the fatigue scores across groups and over time. Only graph measures and fatigue scores that were significantly different between groups or over time were further explored.

## Results

### Participant characteristics

A total of 101 participants (34 BCC + , 32 BCC − and 35 NC) were included. The clinical and demographic characteristics of these participants are summarized in Table 1.

**Table 1:** Demographic and clinical variables of participants

Variable	BCC + (*n* = 34)	BCC − (*n* = 32)	NC (*n* = 35)	*p*
Age at T1 in years (M ± SD)	49.7 ± 9.8	50.8 ± 7.1	49.6 ± 10.3	0.836
Age at T2 in years (M ± SD)	50.6 ± 9.8	52.0 ± 7.4	50.6 ± 10.2	0.777
Estimated IQ	101.3 ± 13.7	104.6 ± 12.8	107.4 ± 12.8	0.160
Educational level [*n* (%)]				
Low	3 (8.8)	5 (15.6)	0 (0)	0.198
Middle	8 (23.5)	8 (25)	8 (22.8)	
High	23 (67.6)	19 (59.4)	27 (77.1)	
Premenopausal [*n* (%)]				
T1	19 (55.9)	16 (50)	17 (48.6)	0.835
T2	1 (2.9)	13 (40.6)	17 (48.6)	** < 0.001**^a, b^
Time between T1 and T2 (days)	332.9 ± 61.5	340.9 ± 43.4	365.3 ± 62.8	0.053
Breast cancer grading^c^ [*n* (%)]				
1	2 (5.9)	11 (34.4)		**0.020**^b^
2	17 (50)	11 (34.4)		
3	11 (32.4)	5 (15.6)		
Side of breast affected [*n* (%)]				
Right	12 (40)	15 (46.9)		0.300
Left	18 (60)	13 (40.6)		
Histology [*n* (%)]				
In-situ carcinoma	0	16 (57.1)		** < 0.001**^b^
Invasive carcinoma	30 (100)	10 (35.7)		
Isolated tumor cells	0	2 (7.1)		
Origin [*n* (%)]				
Ductal	28(93.3)	25(89.3)		0.078
Lobular	2(0.07)	0		
Isolated tumor cells	0	3(0.11)		
Treatment [*n* (%)]				
Radiotherapy	25 (73.5)	19 (59.4)		0.223
Tamoxifen	22 (64.7)	NA		
Type of surgery				
Wide local excision	19 (55.9)	20 (62.5)		0.512
Mastectomy	11 (32.2)	8 (25)		
Chemotherapy complications [*n* (%)]				
No	20 (58.8)	NA		
Yes	11 (32.4)	NA		
Time since chemotherapy(days)	208.5 ± 72.0	NA		

*BCC* + breast cancer patients receiving chemotherapy, *BCC − *breast cancer patients with no chemotherapy, *NC* non-cancer controls, *IQ* intelligence quotient, *NART* dutch version of the national adult reading test, *NA* not applicable

Values indicate mean ± SD unless indicated otherwise

Significance was declared by *p* < 0.05 and significant differences are highlighted in bold

^a^Indicates a significant difference between BCC + and NC

^b^Indicates a significant difference between BCC + and BCC −

^c^Grade 1 refers to well differentiated, 2 moderately differentiated and 3 poorly differentiated

No significant differences were seen between groups with regards to age, estimated IQ, or educational status. At T1, no significant group difference was found in menopausal status, however a significant difference was seen at T2, where post-hoc testing revealed more postmenopausal women in BCC + compared to BCC − and NC [*X*^2^(2, *N* = 100) = 19.3, *p* < 0.001], as expected in women undergoing chemotherapeutic treatment.

### Fatigue and related variables

**Fig. 1:**
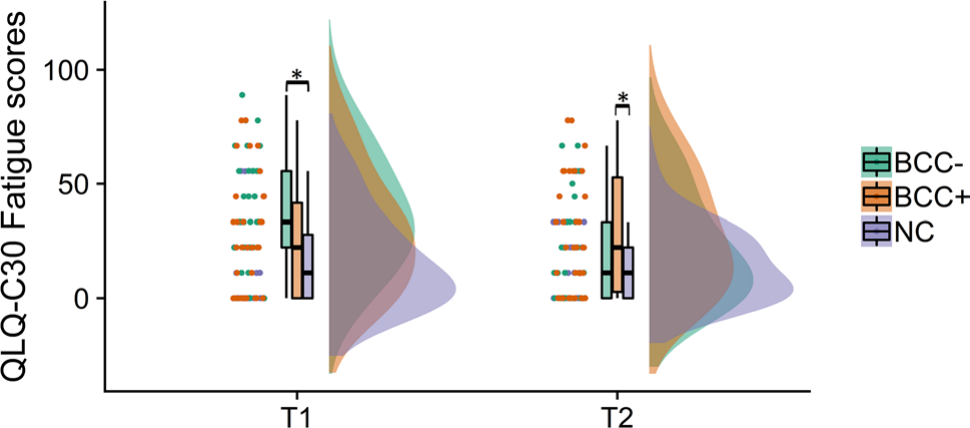
EORTC QLQ-C30 fatigue scores. *BCC − *breast cancer patients with no chemotherapy, *BCC* + breast cancer patients receiving chemotherapy; *NC* non-cancer controls; (See Supplementary Table 2 for more details). *Statistically significant difference defined as *p* < 0.005

A significant difference in EORTC QLQ-C30 fatigue scores was found between the groups at both time points [*F*(2,98) = 7.343, *p* = 0.001 at T1 and *F*(2,97) = 3.936, *p* = 0.023 at T2]. Post-hoc analysis revealed that at T1, the BCC − group reported significantly higher fatigue scores compared to the NC group whereas at T2, the BCC + group reported significantly higher fatigue scores than NC (see Fig. 1 below).

There was also a main effect of time with regards to fatigue [*F*(2,97) = 7.384, *p* = 0.001]; post-hoc analysis group comparison revealed a significant increase in fatigue in the BCC + group (*p* = 0.025), while a significant decrease was seen in the BCC − group (*p* = 0.013) at T2. No significant change was observed in the NC group.

The observed significant difference between BCC − and NC in fatigue scores at T1 was still significant when adjusting for age at measurement, perceived stress score, menopausal status, and HSCL anxiety and depression subscales. However, at T2, the observed significant difference between BCC + and NC lost significance (*p* = 0.456) when adjusted for perceived stress score (*p* = 0.701), menopausal status (*p* = 0.054), HSCL depression score (*p* = 0.067) and HSCL anxiety score (*p* = 0.625). For scores on the PSS and HSCL-25 subscales, please refer to supplementary materials (Supplementary Table 2).

### Network topology

**Fig. 2:**
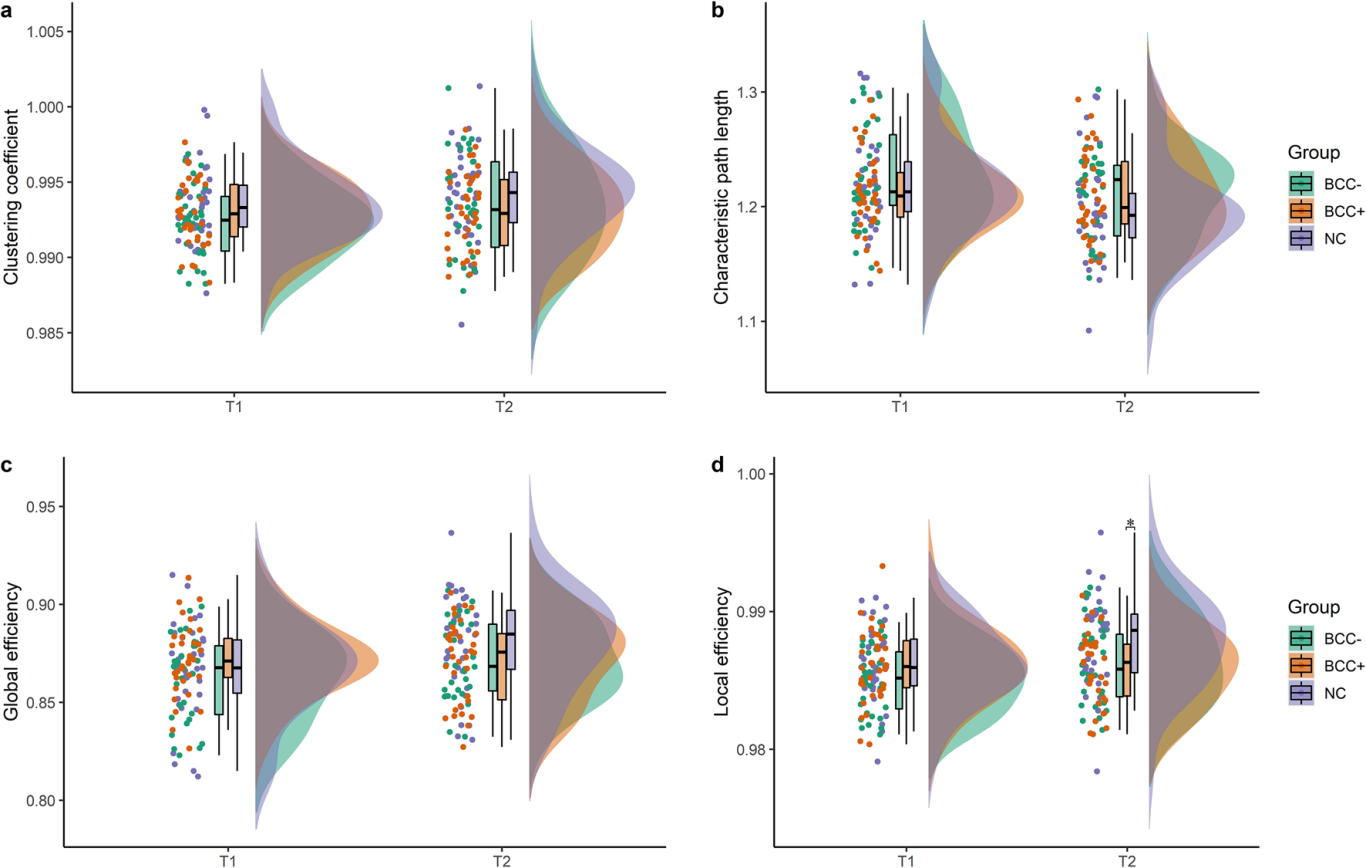
Graph measure values of participants at T1 and T2. These values represent values after normalization by random networks. **A** Clustering coefficient (*C*_p_) values of participants **B** Characteristic path length (*L*_p_) of participants **C** Global efficiency (*E*_glob_) values at T1 and T2 **D** Local efficiency (*E*_loc_) of participants at T1 and T2. *NC* non-cancer control, *BCC − *breast cancer patients without chemotherapy, *BCC* + breast cancer patients with chemotherapy. *Statistically significant difference defined as *p* < 0.05

There was no group × time interaction for any of the graph measures *C*_p_ [*F*(1,98) = 3.428, *p* = 0.067], *L*_p_ [*F*(2,98) = 1.965, *p* = 0.146], *E*_glob_ [*F*(2,98) = 0.968, *p* = 0.384], *E*_loc_ [*F*(2,98) = 2.494, *p* = 0.088)]. Moreover, no significant main effects of group were seen except for *E*_loc_ [*F*(2,98) = 3.150, *p* = 0.047 and see Supplementary Table 4]. Therefore, post-hoc group differences at each time point were further explored for *E*_loc_. There was no statistically significant difference among the three groups at T1, however, at T2 a significant difference was observed [*F*(2,98) = 3.612, *p* = 0.031] (Fig. 2d). Post-hoc analysis revealed that the BCC + group had significantly lower *E*_loc_ values compared to the NC group (*p* = 0.033). (See Table 2, Fig. 2).

**Table 2:** Normalized graph measures at time points T1 and T2

Graph measure		NC (*n* = *35*)	BCC − (*n* = *32*)	BCC + (*n* = *34*)	*p*
*C*_p_	T1	0.993 ± 0.002	0.992 ± 0.002	0.992 ± 0.002	0.996
	T2	0.994 ± 0.003	0.993 ± 0.003	0.993 ± 0.003	0.285
*L*_p_	T1	1.218 ± 0.044	1.226 ± 0.043	1.213 ± 0.035	0.411
	T2	1.196 ± 0.044	1.211 ± 0.037	1.209 ± 0.038	0.207
*E*_loc_	T1	0.986 ± 0.003	0.985 ± 0.002	0.986 ± 0.003	0.151
	T2	0.988 ± 0.003	0.984 ± 0.003	0.986 ± 0.003	**0.031**
*E*_glob_	T1	0.867 ± 0.024	0.862 ± 0.021	0.871 ± 0.018	0.241
	T2	0.880 ± 0.025	0.871 ± 0.019	0.871 ± 0.020	0.477

*BCC − *breast cancer patients with no chemotherapy, *BCC* + breast cancer patients receiving chemotherapy, *NC* non-cancer controls, *C*_p_ clustering coefficient, *L*_p_ characteristic path length, *E*_loc_ local efficiency, *E*_glob_ global efficiency

Values represent mean ± SD. Values were calculated after each participants’ network was normalized with random networks

Significance was declared by *p* < 0.05 and significant differences are highlighted in bold

### Relationship between fatigue and network topology

For all participants as a whole, at both time points a significant negative correlation was observed between fatigue scores and *E*_loc_ (rho = − 0.274, *p* = 0.006 at T1 and rho = − 0.198, *p* = 0.047) (See Table 3). However, analysis on group level showed a significant negative correlation between *E*_loc_ and fatigue scores only in the NC (rho = − 0.34, *p* = 0.026) and BCC − group (rho = − 0.045, *p* = 0.024) at T1, and in the BCC − group (rho = − 0.5, *p* = 0.011) at T2 (See Supplementary Table 3).

**Table 3:** Correlation between fatigue scores and graph measures for all participants

Graph measures	EORTC QLQ-C30 fatigue score			
	T1		T2	
	*rho*	*p*	*rho*	*p*
*C*_p_	− 0.069	0.495	0.001	0.989
*L*_p_	0.191	0.055	0.109	0.277
*E*_glob_	− 0.203	**0**.**041**	− 0.069	0.492
*E*_loc_	− 0.274	**0.006**	− 0.198	**0.047**

*EORTC QLQ-C30* European organization for research and treatment of cancer health-related quality-of-life questionnaire, *C*_p_ clustering coefficient, *L*_p_ characteristic path length, *E*_loc_ local efficiency, *E*_glob_ global efficiency, *rho* Spearman’s rank correlation coefficient

Significance was declared by *p* < 0.05 and significant differences are highlighted in bold

## Discussion

The present study examined fatigue and functional brain network topology of chemotherapy-treated breast cancer patients. The longitudinal design enabled comparisons between and within groups and two control groups were included: one of breast cancer patients who did not receive chemotherapy and one of age-matched non-cancer controls. As expected, patients in the BCC + group reported higher fatigue following chemotherapy than non-cancer controls. Moreover, the BCC + group experienced a significant increase in fatigue between the two time points while fatigue significantly decreased in the BCC- group. Following chemotherapy, differences in brain network topology were observed, with the BCC + group having lower local efficiency compared to the NC group. Finally, a negative association between local efficiency and fatigue scores was seen at both time points in several subgroups.

In line with previous findings [6, 50, 51], greater fatigue was reported in breast cancer patients compared to non-cancer controls. At T1, only BCC − patients reported a significantly higher fatigue score compared to NC. No similar pattern was found in the BCC + group. This finding could be attributed to other comorbidities or personal factors in the BCC − group that were not recorded in this study. Another explanation is that the BCC − patients were in a different emotional stage of cancer diagnosis and treatment compared to the BCC + patients receiving chemotherapy. Previous clinical and anecdotal evidence suggest that the immediate post-treatment phase is characterized by disruption, transition and increased stress which improves over time [52]. The significant decline in fatigue in the BCC- group over time may indicate the emotional progression of patients through the post-treatment stage and their adjustment to life. However, this explanation is speculative, particularly since the EORTC QLQ C-30 fatigue subscale mainly focuses on physical, not mental fatigue. At T2, greater fatigue was reported by the BCC + group compared to NC, where a statistically significant increase in fatigue was also seen between T1 and T2. This increasing level of fatigue can be attributed to chemotherapy, as several previous studies have reported the association between fatigue and chemotherapy in breast cancer patients [53, 54]. Furthermore, the change in menopausal status is a potential contributing factor as the role of premature menopause on prevalence of persistent fatigue in breast cancer patients has been described before [55]. However, cancer-related fatigue is a complex disorder whose pathogenesis is not well understood and the involvement of several psychological and biochemical systems requires further investigation. Our findings regarding comorbid differences between BCC + patients and controls regarding perceived stress, anxiety and depression further support a multidimensional view of fatigue in these patients.

We then investigated the functional brain network of the three different groups. No significant group differences in graph measures were seen at T1, supporting a previous study revealing no differences in global clustering prior to chemotherapeutic treatment [56]. At T2, only differences in *E*_loc_ were seen between the BCC + and NC group, such that the BCC + patients had lower local efficiency than the non-cancer controls, reflecting reduced network segregation. This finding is in line with previous studies showing lower clustering and local efficiency in chemotherapy-treated breast cancer patients as compared to healthy controls at different follow-up durations [31, 32]. However, we are unable to unequivocally conclude that these alterations in functional brain topology are caused by chemotherapy alone as the effect of time was non-significant. Moreover, given that no correction was done for testing of multiple network measures, interpretation of the changes requires careful consideration.

Furthermore, greater fatigue score was correlated with lower local efficiency at both time points, indicating that neural correlates of fatigue may be represented by changes in local information exchange and processing, in contrast to the other graph measures assessed in this study. This shows the significance of network efficiency for fatigue in general, as such correlations were present in both BCC + , BCC − and NC subgroups. Although lower local efficiency is thus not a chemotherapy-specific correlate of fatigue, the robustness of its association across subgroups does suggest that functional brain network topology is a relevant neural correlate of fatigue in any population.

This study has several advantages. The double-controlled, longitudinal design enabled us to determine the added effect of chemotherapy on the brain and fatigue symptoms experienced post-therapy and allowed us to disentangle both specific and general correlates of functional network topology. Extrapolation of brain networks using rsfMRI circumvents the bias associated with task-based fMRI and the use of graph measures allowed quantitative comparison of brain network topology among groups. However, the study also has limitations. Fatigue was measured using the EORTC QLQ-C30 scale, which mainly measures physical fatigue. Given its multidimensional nature, the use of domain-specific scales is important towards greater understanding of cancer-related fatigue [57, 58]. Also, baseline assessments were done after surgery under general anesthesia and the contribution of anesthetic agents to the observed change cannot be excluded. In addition, a subset of breast cancer patients underwent mastectomy; we did not investigate the effect of mastectomy on fatigue in this study. The generalization of our findings to all breast cancer patients should take the association between mastectomy and mental fatigue reported by some studies [59, 60] into consideration. Furthermore, it should be noted that 65% of the BCC + participants received tamoxifen therapy, whose effect on the brain is controversial and largely under investigated [61]. Due to the lack of adequate sample size we were not able to assess the specific effect of tamoxifen on brain networks in this study.

In summary, to the best of our knowledge, this is the first longitudinal study with two control groups investigating fatigue, resting-state functional brain networks and their association in breast cancer patients. The longitudinal design allowed for assessment of the potentially specific effects of chemotherapy on fatigue and functional brain network topology. The two control groups provided comparisons within breast cancer patients and with age-matched non-cancer controls. Our study shows the differences in network topology and fatigue among the groups following chemotherapy. Breast cancer patients exhibited increased fatigue and decreased local information processing and exchange following chemotherapy. However, a clear causal association between changes in brain topology and chemotherapy is yet to be established. Furthermore, a general correlation between network topology and fatigue across all groups was seen, for the first time indicating a general neural correlate in this population. Further studies using more sensitive fatigue measurement scales are required to better characterize fatigue experienced by breast cancer patients and its associations with brain functioning. Assessments further in time are also crucial in determining the persistence of changes and long-term effects of systemic cancer treatment on the brain.

## Supplementary Information

Below is the link to the electronic supplementary material.

Supplementary file1 (DOCX 29 kb)

## Data Availability

The datasets generated during and/or analysed during the current study are available from the corresponding author on reasonable request.

## Abbreviations

A: Doxorubicin
AAL: Automated anatomical labeling
BCC −: Breast cancer patients not treated with chemotherapy
BCC +: Breast cancer patients treated with chemotherapy
BOLD: Blood oxygen level dependent
C: Cyclophosphamide
*C*_p_: Clustering coefficient
DMN: Default mode network
E: Epirubicin
*E*_glob_: Global efficiency
*E*_loc_: Local efficiency
EORTC QLQ-C30: European organization for research and treatment of cancer quality of life questionnaire-C30
EPI: Echo planar imaging
F: 5-Flurouracil
fMRI: Functional magnetic resonance imaging
HSCL-25: Hopkins symptoms checklist-25
ICA-AROMA: Independent component analysis-automatic removal of motion artifacts
*L*_p_: Characteristic path length
NART: Dutch adult reading test
NC: Non-cancer control
PROs: Patient reported outcomes
PSS: Perceived stress scale
rsfMRI: Resting-state functional magnetic resonance imaging
SED: Standard error of measurement of the difference
T: Docetaxel

## Code availability

Code for analysis is available upon reasonable request.
