# Supplementary material for: Fatigue and resting-state functional brain networks in breast cancer patients treated with chemotherapy
Source: Breast Cancer Res Treat. 2021 Jul 14;189(3):787–96. doi: 10.1007/s10549-021-06326-0 (PMC8505321; doi:10.1007/s10549-021-06326-0)
Supplement: Supplementary file 1 — Supplementary file1 (DOCX 29 kb) [file 10549_2021_6326_MOESM1_ESM.docx]

Breast Cancer Research and Treatment

**Fatigue and resting-state functional brain networks in breast cancer patients treated with chemotherapy**

Biniam Melese Bekele,^a,b^ Maryse Luijendijk,^b,c^ Sanne B. Schagen,^b,c^ Michiel de Ruiter,^b^ Linda Douw^a,*^

^a^ Department of Anatomy and Neurosciences, Amsterdam University Medical Centers, Vrije Universiteit Amsterdam, Amsterdam Neuroscience, Cancer Center Amsterdam, Amsterdam, The Netherlands

^b^ Department of Psychosocial Research and Epidemiology, Netherlands Cancer Institute, Amsterdam, The Netherlands

^c^ Brain and Cognition, Department of Psychology, University of Amsterdam, Amsterdam, The Netherlands

^*^Corresponding author at Department of Anatomy and Neurosciences, Amsterdam University Medical Centers, Vrije Universiteit Amsterdam, Amsterdam Neuroscience, Cancer Center Amsterdam, Amsterdam, The Netherlands, [l.douw@amsterdamumc.nl](mailto:l.douw@amsterdamumc.nl)

# Supplementary materials

**Supplementary Table 1: Chemotherapy regimens**

| **Chemotherapy** | **Regimen description** | ***n (%)*** |
| --- | --- | --- |
| AC | Four or six cycles of A and C | 3 (8.8)  22 (64.6)  6 (17.6)  3 (8.8) |
| TAC | Six cycles of T, A and C |  |
| AC-paclitaxel | Four cycles of AC followed by four or twelve cycles of paclitaxel |  |
| FEC | Three or six cycles of F, E and C |  |
| A – Doxorubicin, C – Cyclophosphamide; E – Epirubicin; F – 5-flurouracil; T – Docetaxel  *n (%)* – indicate the number (proportion from total BCC+) of participants who received the specific chemotherapy regimen | | |

**Supplementary Table 2: Selected subscales of patient reported outcomes**

| **Questionnaire** | | **BCC+ (*n = 34*)** | **BCC- (*n = 32*)** | **NC (*n = 35*)** | ***p***^1^ |
| --- | --- | --- | --- | --- | --- |
| QLQ-C30:Fatigue^2^ | |  |  |  |  |
| T1 | | 27.4 ± 24.5 | 36.8 ± 24.5 | 15.6 ± 19.1 | **0.001** ^a^ |
| T2 | | 28.3 ± 25.0 | 20.7 ± 22.3 | 14.0 ± 14.8 | **0.023**^b^ |
| HSCL-25^3^ | |  |  |  |  |
| Total score | |  |  |  |  |
|  | T1 | 11.8 ± 9.8 | 12.5 ± 11.6 | 5.7 ± 5.8 | **0.006** ^a, b^ |
|  | T2 | 10.6 ± 12.6 | 10.1 ± 10.7 | 4.8 ± 5.1 | **0.029**^b^ |
| Anxiety | |  |  |  |  |
|  | T1 | 11.6 ± 8.5 | 9.8 ± 9.3 | 5.1 ± 5.8 | **0.003** ^b^ |
|  | T2 | 11.1 ± 10.8 | 8.4 ± 8.6 | 5.1 ± 5.7 | **0.016**^b^ |
| Depression | |  |  |  |  |
|  | T1 | 12.0 ± 11.8 | 14.2 ± 15.2 | 6.2 ± 7.3 | **0.017** ^a^ |
|  | T2 | 10.3 ± 14.6 | 11.2 ± 13.7 | 4.6 ± 5.8 | 0.05 |
| PSS^4^ | |  |  |  |  |
| T1 | | 24.2 ± 5.9 | 20.5 ± 7.7 | 18.6 ± 4.8 | **0.001**^b^ |
| T2 | | 20.2 ± 5.0 | 20.3 ± 5.8 | 18.6 ± 4.9 | 0.306 |
| BCC+ – Breast cancer patients receiving chemotherapy; BCC- – Breast cancer patients with no chemotherapy; NC – Non-cancer controls; QLQ-C30 – European Organization for Research and Treatment of Cancer Health-Related Quality-of-Life Questionnaire; HSCL-25 – Hopkins Symptoms Checklist-25; PSS – Perceived Stress scale  Values indicate mean + SD unless indicated otherwise. Significance is defined as *P* < 0.05  ^1^ – One-way ANOVA test with df=2  ^2^ – Scores range from 0 to 100, Higher scores indicate more fatigue  ^3^ – Scores range from 0 to 100, Higher scores indicate higher levels of anxiety and depression  ^4^ – Scores range from 10 to 50, Higher scores indicate higher levels of perceived stress  ^a^ – indicates a significant difference between BCC- and NC  ^b^ – indicates a significant difference between BCC+ and NC  ^c^ – indicates a significant difference between BCC+ and BCC- | | | | | |

**Supplementary Table 3: Group level correlation between fatigue scores and graph measures**

| **Groups** | **Graph measures** | **EORTC QLQ-C30 fatigue score** | | | |
| --- | --- | --- | --- | --- | --- |
|  |  | **T1** | | **T2** | |
|  |  | ***rho*** | ***p*** | ***rho*** | ***p*** |
| NC | Cp | 0.09 | 0.439 | -0.2 | 0.543 |
|  | Lp | 0.28 | **0.009** | -0.1 | 0.956 |
|  | E_glob_ | -0.25 | **0.013** | -0.05 | 0.705 |
|  | E_loc_ | -0.34 | **0.026** | 0.06 | 0.715 |
| BCC- | Cp | -0.27 | 0.346 | -0.11 | 0.357 |
|  | Lp | 0.37 | 0.062 | 0.33 | 0.135 |
|  | E_glob_ | -0.36 | 0.072 | -0.49 | **0.016** |
|  | E_loc_ | -0.45 | **0.024** | -0.5 | **0.011** |
| BCC+ | Cp | 0.16 | 0.949 | 0.23 | 0.570 |
|  | Lp | -0.17 | 0.314 | -0.01 | 0.956 |
|  | E_glob_ | 0.1 | 0.482 | 0.44 | **0.034** |
|  | E_loc_ | 0.04 | 0.633 | 0.05 | 0.938 |
| BCC+ – Breast cancer patients receiving chemotherapy; BCC- – Breast cancer patients with no chemotherapy; NC – Non-cancer controls; EORTC QLQ-C30 – European Organization for Research and Treatment of Cancer Health-Related Quality-of-Life Questionnaire; Cp – Clustering coefficient, Lp – Characteristic path length, E_loc_ – Local efficiency, E_glob_ – Global efficiency; rho – Spearman’s rank correlation coefficient | | | | | |

**Supplementary Table 4: Repeated-measure ANOVA of graph measures**

| **Graph measure** | **Variable** | **df** | **F** | ***p*** |
| --- | --- | --- | --- | --- |
| Cp | Group | 2 | 2.157 | 0.121 |
|  | Time | 1 | 3.428 | 0.067 |
|  | Time*Group | 2 | 0.668 | 0.515 |
| Lp | Group | 2 | 0.974 | 0.381 |
|  | Time | 1 | 10.92 | **0.001** |
|  | Time*Group | 2 | 1.965 | 0.146 |
| E_glob_ | Group | 2 | 0.580 | 0.562 |
|  | Time | 1 | 4.324 | **0.04** |
|  | Time*Group | 2 | 0.968 | 0.384 |
| E_loc_ | Group | 2 | 3.150 | **0.047** |
|  | Time | 2 | 7.675 | **0.007** |
|  | Time*Group | 2 | 2.494 | 0.088 |
| Cp – Clustering coefficient, Lp – Characteristic path length, E_loc_ – Local efficiency, E_glob_ – Global efficiency; df – Degrees of freedom | | | | |
